# Phenotypic, Functional, and Gene Expression Profiling of Peripheral CD45RA+ and CD45RO+ CD4+CD25+CD127^low^ Treg Cells in Patients With Chronic Rheumatoid Arthritis

**DOI:** 10.1002/art.39408

**Published:** 2015-12-23

**Authors:** Gina J. Walter, Veerle Fleskens, Klaus S. Frederiksen, Megha Rajasekhar, Bina Menon, Jens G. Gerwien, Hayley G. Evans, Leonie S. Taams

**Affiliations:** ^1^ King's College LondonLondonUK; ^2^ Novo NordiskMåløvDenmark; ^3^ Guy's and St. Thomas’ NHS Foundation TrustLondonUK

## Abstract

**Objective:**

Conflicting evidence exists regarding the suppressive capacity of Treg cells in the peripheral blood (PB) of patients with rheumatoid arthritis (RA). The aim of this study was to determine whether Treg cells are intrinsically defective in RA.

**Methods:**

Using a range of assays on PB samples from patients with chronic RA and healthy controls, CD3+CD4+CD25+CD127^low^ Treg cells from the CD45RO+ or CD45RA+ T cell compartments were analyzed for phenotype, cytokine expression (ex vivo and after in vitro stimulation), suppression of Teff cell proliferation and cytokine production, suppression of monocyte‐derived cytokine/chemokine production, and gene expression profiles.

**Results:**

No differences between RA patients and healthy controls were observed with regard to the frequency of Treg cells, ex vivo phenotype (CD4, CD25, CD127, CD39, or CD161), or proinflammatory cytokine profile (interleukin‐17 [IL‐17], interferon‐γ [IFNγ], or tumor necrosis factor [TNF]). FoxP3 expression was slightly increased in Treg cells from RA patients. The ability of Treg cells to suppress the proliferation of T cells or the production of cytokines (IFNγ or TNF) upon coculture with autologous CD45RO+ Teff cells and monocytes was not significantly different between RA patients and healthy controls. In PB samples from some RA patients, CD45RO+ Treg cells showed an impaired ability to suppress the production of certain cytokines/chemokines (IL‐1β, IL‐1 receptor antagonist, IL‐7, CCL3, or CCL4) by autologous lipopolysaccharide‐activated monocytes. However, this was not observed in all patients, and other cytokines/chemokines (TNF, IL‐6, IL‐8, IL‐12, IL‐15, or CCL5) were generally suppressed. Finally, gene expression profiling of CD45RA+ or CD45RO+ Treg cells from the PB revealed no statistically significant differences between RA patients and healthy controls.

**Conclusion:**

Our findings indicate that there is no global defect in either CD45RO+ or CD45RA+ Treg cells in the PB of patients with chronic RA.

T cells with a regulatory phenotype (i.e., CD4+CD25+CD127^low^FoxP3+) are abundantly present in the inflamed joints of patients with rheumatoid arthritis (RA) 1, 2, 3, 4, 5, 6, 7, 8. However, despite their presence, inflammation persists, thus posing the question as to whether Treg cells are functionally impaired in RA. Evidence that CD4+CD25+ Treg cells are important in controlling the severity of arthritis comes from experimental mouse studies in which depletion of Treg cells using an anti‐CD25–depleting antibody before immunization resulted in exacerbated disease 9, 10. Conversely, adoptive transfer of CD4+CD25+ Treg cells in the early phase of the disease led to a reduction in disease severity 10, 11. Additionally, earlier onset of disease and more aggressive disease progression were observed in the K/BxN model of spontaneous arthritis in scurfy mice, a mouse strain that is devoid of Treg cells due to a mutation in the *Foxp3* gene and, consequently, develops severe multiorgan inflammation 12. These data suggest that a functional impairment of Treg cells may contribute to chronic joint inflammation.

Indeed, several groups of investigators have shown that peripheral Treg cell function is defective in RA patients 13, 14, 15, 16. It was reported that Treg cells from patients with active RA can suppress the proliferation of Teff cells, but the ability of Treg cells to inhibit proinflammatory cytokine production, such as production of interferon‐γ (IFNγ) and tumor necrosis factor (TNF) by T cells and production of TNF by monocytes, is impaired 13. The inability of Treg cells from RA patients to suppress IFNγ production in Teff cells has also been demonstrated by other groups 15, 16, 17. It was proposed that this functional defect may be caused by negative effects of TNF on Treg cell function 14, 15, which was supported by the finding that TNF blockade could improve Treg cell function 13, 14, 15, 18.

However, results from several studies have contradicted the notion that defective Treg cell function contributes to inflammatory arthritis. In nude mice injected with CD25‐depleted lymphocyte suspensions, relatively few animals developed signs of polyarthritis under non–disease‐inducing conditions 19, 20. In addition, in human studies, signs of arthritis were observed in only a few cases of X‐linked syndrome of immune dysregulation, polyendocrinopathy, and enteropathy (IPEX), a disease that develops in individuals with a *FOXP3* gene mutation 21, 22; instead, patients with IPEX present with thrombocytopenia, insulin‐dependent diabetes mellitus, diarrhea, or thyroiditis 22. These findings suggest that there is no direct correlation between impaired Treg cell presence and/or function and the development of arthritis.

Furthermore, several groups, including our own, have shown that Treg cells from the peripheral blood (PB) of patients with RA are intact in their capacity to suppress the proliferation of, or cytokine production by, Teff cells 2, 3, 5, 7, 23, 24. Moreover, in all studies except one 14 that have investigated CD4+CD25+ Treg cells in the inflamed joints of patients with arthritis, the findings concur, showing that these cells are functionally intact and are fully capable of suppressing proliferation and cytokine production ex vivo 1, 2, 3, 4, 5, 6, 7, 25, 26, 27. Instead, the dysregulation of Teff cell function in arthritis may be attributed to resistance of activated Teff cells to Treg cell–mediated suppression, rather than to intrinsic defects in Treg cell function 2, 26, 28, 29, 30, 31.

Reasons for the conflicting results obtained in the studies investigating PB‐derived Treg cells in RA patients are neither well reported nor understood, but appear to not be simply explained by differences in cell isolation procedures or patient characteristics (for review, see ref. 32). Interpretation of the results may, however, be restricted by the fact that most of these earlier studies utilized only a limited range of readout assays, often focusing on suppression of either Teff cell proliferation or cytokine production (mostly, production of IFNγ, TNF, or interleukin‐17 [IL‐17]). We sought to shed light on whether PB Treg cells are truly dysfunctional in RA by performing an extensive analysis of CD3+CD4+CD25+CD127^low^ cells (hereafter referred to as Treg cells) in both the CD45RO+ and CD45RA+ T cell compartments, using a wide range of readout systems. Our collective data indicate that there is no global defect in either CD45RO+ memory Treg cells or CD45RA+ naive Treg cells in the PB of patients with chronic RA.

## PATIENTS AND METHODS

### Patients and healthy volunteers

PB and synovial fluid (SF) samples were obtained from patients with RA recruited from the rheumatology outpatient clinic at Guy's and St. Thomas’ Hospital NHS Trust (London, UK). Clinical parameters such as Disease Activity Score in 28 joints (DAS28) 33, erythrocyte sedimentation rate (ESR), C‐reactive protein (CRP) level, rheumatoid factor status, medication, age, sex, and disease duration were documented on the day of sample collection (Table 1). Healthy control subjects were recruited from among local staff volunteers. Written informed consent was received from all participants. Samples were collected in compliance with the Declaration of Helsinki. Ethics approval was given by the Bromley Research Ethics Committee (approval no. 06/Q0705/20).

**Table 1:** Demographic and clinical characteristics of the patients with rheumatoid arthritis (RA) and healthy control subjects^a^

	Treg cell frequency and phenotype assays			Functional assays	
	RA PB (n = 43)	RA SF (n = 13)	Healthy controls (n = 42)	RA PB (n = 15)	Healthy controls (n = 12)
Sex, no. female/no. male	38/5	11/2	27/15	11/4	9/3
Age, years					
Mean ± SEM	56 ± 2.2	59 ± 4.5	37 ± 2.2	50 ± 2.4	43 ± 4.1
Range	25–89	26–89	18–69	31–64	22–60
DAS28					
Mean ± SEM	5.0 ± 0.2	4.8 ± 0.3	–	4.9 ± 0.3	–
Range	2.6–6.9	2.6–6.9	–	2.7–6.9	–
ESR, mm/hour					
Median (IQR)	16 (9.5–33)	22 (16–37)	–	18 (12–50)	–
Range	2.0–75	7.0–75	–	2.0–75	–
CRP, mg/liter					
Median (IQR)	6.0 (5.0–15.3)	15 (7.5–18)	–	11 (5.0–30)	–
Range	5.0–43	5.0−40	–	5.0–64	–
Treatment, no. of patients					
None	4	3	–	1	–
NSAID	2	2	–	0	–
DMARD^b^	33	1	–	12	–
TNF inhibitor	3	7	–	2	–
Steroids	1	0	–	0	–
RF, no. positive/no. negative	32/8	12/1	–	12/2	–
Disease duration, years					
Median (IQR)	5.7 (2.3–18)	7.5 (2.0–20)	–	4.0 (1.8–6.5)	–
Range	0–60	0–60	–	0.5–40	–

a In these analyses, data from the peripheral blood (PB) or synovial fluid (SF) of RA patients were not available for some of the variables, as follows: in the Treg cell frequency and phenotype assays, n = 1 for the Disease Activity Score in 28 joints (DAS28), n = 2 for erythrocyte sedimentation rate (ESR), n = 5 for C‐reactive protein (CRP) level and n = 12 with a CRP level <5 mg/liter, n = 3 for rheumatoid factor (RF) status, and n = 7 RA PB and n = 3 RA SF for disease duration; in the functional assays, n = 1 for age, n = 1 for ESR, n = 2 for CRP level and n = 2 with a CRP level <5 mg/liter, n = 1 for RF status, and n = 2 for disease duration. IQR = interquartile range; TNF = tumor necrosis factor.

b Disease‐modifying antirheumatic drug (DMARD) use was as follows: in patients analyzed for Treg cell frequency and phenotype, methotrexate (MTX) n = 15, hydroxychloroquine (HCQ) n = 3, sulfasalazine (SSZ) n = 2, MTX/HCQ n = 4, MTX/SSZ n = 2, MTX/HCQ/SSZ n = 4, MTX/HCQ/steroid n = 1, MTX/nonsteroidal antiinflammatory drug (NSAID) n = 1, and HCQ/SSZ n = 1; in patients analyzed for Treg cell function, MTX n = 8, SSZ n = 1, MTX/HCQ n = 1, MTX/HCQ/SSZ n = 1, and MTX/steroid n = 1.

### Cell isolation

PB mononuclear cells (PBMCs) and SF mononuclear cells were isolated by Ficoll‐Hypaque (LSM 1077; PAA Laboratories) density‐gradient centrifugation. CD14+ monocytes (purity >98%) were positively selected using CD14 MicroBeads (Miltenyi Biotec). CD4+ T cells were isolated from the CD14− cell fraction by negative selection (Miltenyi Biotec) and stained with PerCP/Cy5.5‐conjugated CD4, allophycocyanin (APC)/Cy7–conjugated CD45RA, Pacific Blue–conjugated CD45RO, fluorescein isothiocyanate (FITC)–conjugated CD127 (BioLegend), and phycoerythrin (PE)–conjugated CD25 (Miltenyi Biotec). CD4+CD45RA+CD45RO−CD25+CD127^low^ cells (naive Treg cells), CD4+CD45RA−CD45RO+CD25+CD127^low^ cells (memory Treg cells), CD4+CD45RA−CD45RO+CD25−CD127+ cells (CD25− memory Teff cells), or CD4+CD45RA−CD45RO+CD25^intermediate^CD127+ cells (CD25^int^ memory Teff cells) were sorted by fluorescence‐activated cell sorting (FACS) analysis using a BD FACSAria II (>98% purity for each subset) (see Supplementary Figure 1, available on the *Arthritis & Rheumatology* web site at http://onlinelibrary.wiley.com/doi/10.1002/art.39408/abstract).

**Figure 1:**
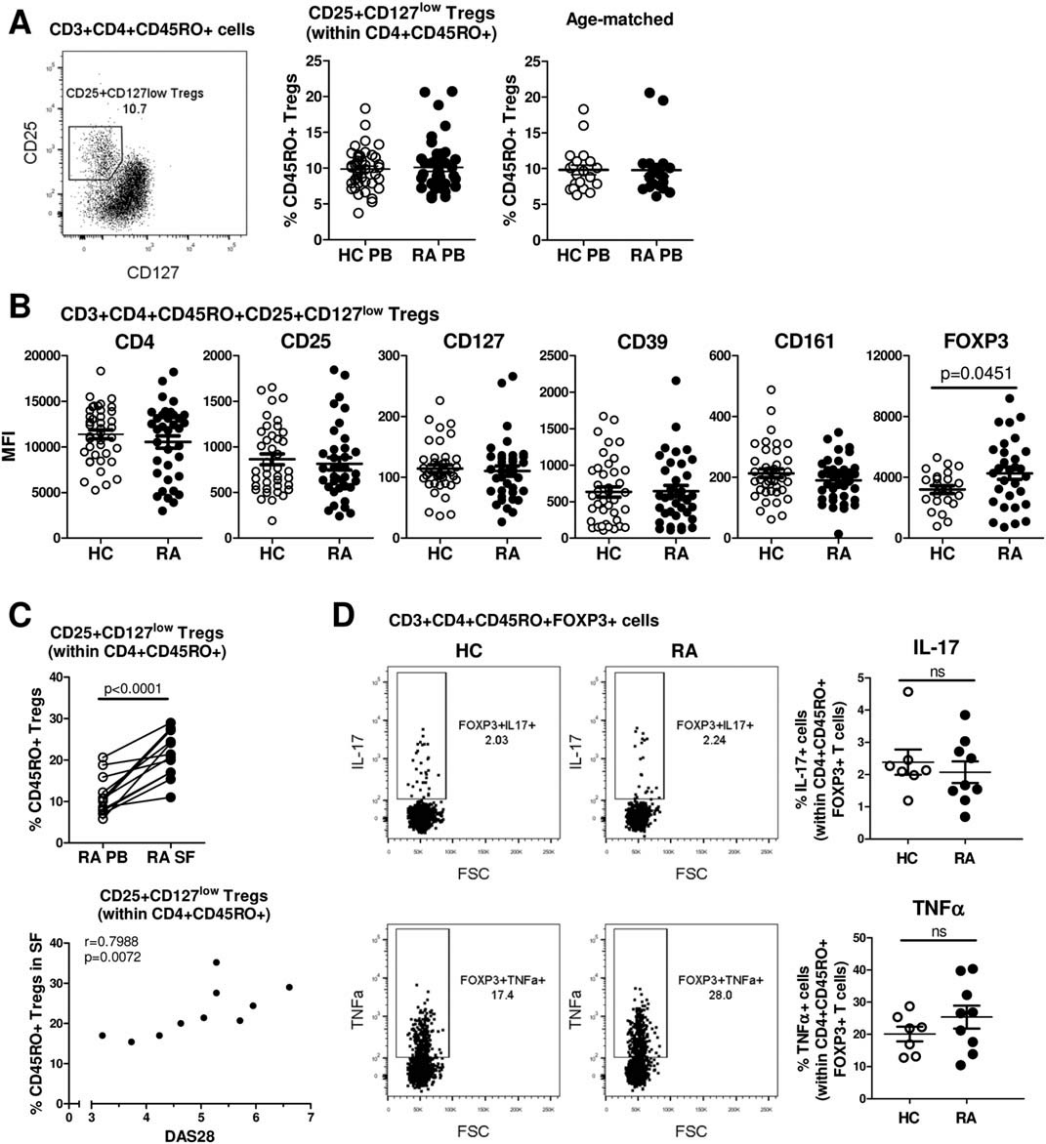
Frequency and phenotype of CD3+CD4+CD45RO+CD25+CD127^low^ Treg cells in patients with rheumatoid arthritis (RA). **A,** Fluorescence‐activated cell sorting analysis was used to gate on CD25+CD127^low^ Treg cells (boxed area with percentage value shown) within the CD4+CD45RO+ T cell population in peripheral blood mononuclear cells (PBMCs) from RA patients (left). Percentages of these cells were compared between healthy controls (HC) and RA patients (each n = 42) (center) or between age‐matched healthy controls (mean ± SD age 49 ± 2.5 years) and RA patients (mean ± SD age 49 ± 2.4 years) (each n = 20) (right). **B,** Expression levels of the indicated surface markers in CD3+CD4+CD45RO+CD25+CD127^low^ Treg cells were analyzed in PBMCs from healthy controls (n = 40) and RA patients (n = 36). FoxP3 expression was measured intracellularly (n = 23 healthy controls, n = 30 RA patients). **C,** The percentage of CD25+CD127^low^ cells within CD4+CD45RO+ T cells was determined in paired samples of freshly isolated PB and synovial fluid (SF) from RA patients (n = 15) (top), and Treg cell frequencies in the RA SF (n = 10) were assessed for correlation with the Disease Activity Score in 28 joints (DAS28), by Spearman's test (bottom). Symbols represent individual patients. **D,** PBMCs from healthy controls (n = 7) and RA patients (n = 9) were stimulated with phorbol myristate acetate and ionomycin for 3 hours in the presence of GolgiStop, and the percentage of interleukin‐17 (IL‐17)–positive or tumor necrosis factor (TNF)–positive cells (boxed areas with percentage values shown) within CD3+CD4+CD14−CD45RO+FoxP3+ T cells was determined. Representative dot plots are shown. In **A**, **B,** and **D**, symbols represent individual donors; horizontal bars show the mean. Groups were compared by either paired or unpaired *t*‐test. MFI = mean fluorescence intensity.

### Phenotype and cell frequency analyses

For analyses of phenotype and frequency of each cell subset, PE‐conjugated CD25 (Miltenyi Biotec), APC/Cy7‐conjugated CD3, PerCP/Cy5.5‐conjugated CD4, PE/Cy7‐conjugated CD39, Alexa Fluor 488–conjugated CD127, Alexa Fluor 647–conjugated CD161, Pacific Blue–conjugated CD45RO, and Alexa Fluor 647–conjugated FoxP3 (all from BioLegend) were used. For FoxP3 staining, cells were extracellularly stained, and were then fixed and permeabilized using 1× FoxP3 Perm buffer (BioLegend). Cells were acquired on a BD FACSCanto II and analyzed using FlowJo software (version 7.6.1; Tree Star).

### Analysis of cytokine‐expressing Treg cells

#### Ex vivo culture

PBMCs (2 × 10^6^ cells/ml) were stimulated in RPMI 1640 culture medium containing 20 m*M* l‐glutamine (Gibco), 1% penicillin–streptomycin (Gibco), and 10% fetal bovine serum (batch no. F9665, lot no. 030M3399; Sigma) for 3 hours with phorbol myristate acetate (PMA) (50 ng/ml; Sigma) and ionomycin (750 ng/ml; Sigma) in the presence of GolgiStop (BD Biosciences), in accordance with the manufacturer's instructions. Surface staining for PE/Cy7‐conjugated CD3, APC/Cy7‐conjugated CD14 (BioLegend), and Pacific Blue–conjugated CD45RO was followed by intracellular staining for PerCP/Cy5.5‐conjugated CD4, PE‐conjugated IL‐17A (BioLegend), FITC‐conjugated TNF (BioLegend), or Alexa Fluor 647–conjugated FoxP3.

#### In vitro culture

CD14+ monocytes (1 × 10^5^) were cocultured with sorted Treg cells or Teff cells for 3 days at a 1:1 ratio in culture medium containing 100 ng/ml soluble anti‐CD3 monoclonal antibodies (mAb) (OKT‐3; Janssen‐Cilag) in 96‐well U‐bottomed culture plates at 37°C in an atmosphere of 5% CO_2._ When indicated, 100 ng/ml lipopolysaccharide (LPS; Sigma) was added on day 0. On day 3, the cells were stimulated with PMA/ionomycin for 6 hours, with GolgiStop present for the last 3 hours, followed by surface staining for Pacific Blue–conjugated CD2 (BioLegend) and APC/Cy7‐conjugated CD14. Cells were fixed with 2% paraformaldehyde and intracellularly stained for PE‐conjugated IL‐17A, PerCP/Cy5.5‐conjugated IFNγ, APC‐conjugated TNF, and Alexa Fluor 488–conjugated IL‐10 (all from BioLegend) using 0.5% saponin.

### T cell suppression assay

Freshly sorted CD25− Teff cells (2–5 × 10^6^/ml) or, where indicated, CD25^int^ Teff cells were labeled with 2 μ*M* 5,6‐carboxyfluorescein succinimidyl ester (CFSE; Molecular Probes) for 15 minutes at 37°C. Labeled cells (5 × 10^4^) were cultured with sorted Treg cells at various concentrations (1 × 10^4^−5 × 10^4^) and 5 × 10^4^ monocytes, in the presence or absence of 100 ng/ml LPS, in a 96‐well U‐bottomed plate. On day 3, supernatants were collected for analysis of cytokine production. In addition, T cell proliferation was determined by flow cytometry, measuring CFSE fluorescence. Results are expressed as the percentage suppression of T cell proliferation, calculated using the following formula: (100 − [% proliferation with Treg cells/% proliferation without Treg cells]) × 100.

### Monocyte suppression assay

Freshly isolated CD14+ monocytes (1 × 10^5^) were cultured with or without sorted CD4+CD45RO+CD25+CD127^low^ Treg cells at a 1:1 ratio in the presence of 100 ng/ml LPS and 100 ng/ml anti‐CD3. Supernatants were collected on day 3 for analysis by multiplex assay.

### Cytokine detection

Supernatants were stored at −80°C until analyzed by enzyme‐linked immunosorbent assay (for IL‐6 and IL‐8; BioLegend) or by the Human Cytokine 25‐plex assay (Invitrogen Life Technologies) on the Luminex platform.

### Gene expression profiling

Sorted Treg and Teff cell samples were lysed in 500 μl of TRIzol (Invitrogen). Chloroform (100 μl) was added, and the samples were then whirl mixed and incubated for 2–3 minutes at room temperature. Following centrifugation (10,000*g* for 15 minutes at 4ºC), the water phase was further purified using the RNeasy MinElute Cleanup kit (Qiagen). RNA integrity was confirmed on an Agilent Technologies 2100 bioanalyzer using total RNA Nano chips. One hundred nanograms of total RNA was used to prepare the targets, using the 3 ′ IVT Express kit (Affymetrix) in accordance with the manufacturer's instructions. Hybridization cocktails were hybridized onto a Human Genome U219 Array plate in a GeneTitan instrument (both from Affymetrix). Chips were scanned and gene expression data were normalized using the RMA algorithm and Bioconductor package “Affy” (available at http://www.bioconductor.org). A custom chip definition file (available at http://brainarray.mbni.med.umich.edu) was used. Gene expression profiling analysis was performed using Qlucore Omics Explorer software, version 3.0. The microarray data have been deposited in the GEO database (GEO accession no. GSE65010).

### Statistical analysis

Significance testing was performed with GraphPad Prism software, version 5. Data were tested for normality using the D'Agostino and Pearson omnibus normality test, followed by appropriate parametric or nonparametric testing for significance.

## RESULTS

### Comparing frequencies of PB CD4+CD25+CD127^low^ Treg cells between RA patients and healthy controls

Frequencies of CD4+CD25+CD127^low^ Treg cells 34 in the PB from patients with RA were determined within the CD3+CD4+CD45RO+ (memory) T cell compartment, since CD4+CD25^high^ Treg cells are predominantly memory cells 35 and because T cells in the SF are of the CD45RO+ memory phenotype 36. We found no significant differences in the percentage of CD25+CD127^low^ Treg cells within CD4+CD45RO+ T cells between PB samples from RA patients and those from healthy controls (each n = 42), even when the groups were age‐matched (mean ± SD age 49 ± 2.4 years in RA patients versus 49 ± 2.5 years in healthy controls [each n = 20]) (Figure 1A). Treg cell frequencies did not correlate with age in either the RA cohort or the healthy control cohort (see Supplementary Figure 2A, available on the *Arthritis & Rheumatology* web site at http://onlinelibrary.wiley.com/doi/10.1002/art.39408/abstract). No sex‐specific differences in Treg cell frequencies were found (results not shown). In addition, the frequencies of Treg cells within CD3+CD4+CD45RO− T cells (naive Treg cells) were also similar between RA patients and healthy controls (see Supplementary Figure 2B, available on the *Arthritis & Rheumatology* web site at http://onlinelibrary.wiley.com/doi/10.1002/art.39408/abstract).

We investigated the ex vivo phenotype of CD25+CD127^low^ cells within the CD3+CD4+CD45RO+ T cell compartment in PBMCs from RA patients and healthy controls. CD45RO+ Treg cells from RA patients (n = 36) showed similar expression levels of CD4, CD25, and CD127 as those from healthy controls (n = 40) (Figure 1B). We found no significant differences in the expression levels or the percentage of cells expressing the ectoenzyme CD39, a Treg cell–specific surface marker 37, or CD161, which identifies a subpopulation of Treg cells with increased potential to express the proinflammatory cytokine IL‐17 38 (Figure 1B and Supplementary Figure 2C, available on the *Arthritis & Rheumatology* web site at http://onlinelibrary.wiley.com/doi/10.1002/art.39408/abstract). A small increase in FoxP3 expression was observed in CD45RO+ Treg cells from RA patients (Figure 1B). The phenotype of CD45RO− Treg cells from RA PB showed a very similar pattern as that seen in CD45RO+ Treg cells and was not different from healthy control CD45RO− Treg cells (see Supplementary Figures 2C and D, available on the *Arthritis & Rheumatology* web site at http://onlinelibrary.wiley.com/doi/10.1002/art.39408/abstract).

**Figure 2:**
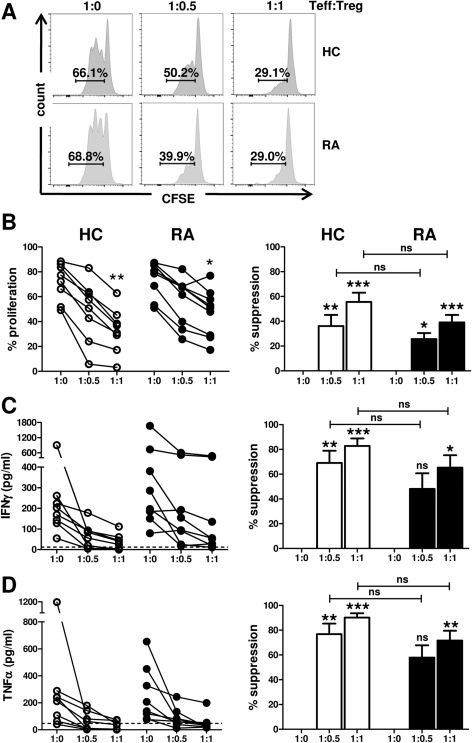
Ability of CD45RO+ Treg cells from patients with RA to suppress Teff cell proliferation and cytokine production. CD4+CD45RA−CD45RO+CD25−CD127+ Teff cells from healthy controls and RA patients were labeled with 5,6‐carboxyfluorescein succinimidyl ester (CFSE) and cocultured with CD14+ monocytes at a 1:1 cell ratio in the presence of anti‐CD3 monoclonal antibody (100 ng/ml), in the absence or presence of autologous sorted CD45RO+ Treg cells (added at the indicated Teff cell:Treg cell ratios). On day 3, cell proliferation was assessed by flow cytometry **(A** and **B)**, and cell culture supernatants were collected for detection of secretion of interferon‐γ (IFNγ) **(C)** and TNF **(D)** by Luminex. **A,** Histograms show the CFSE dilution and the percentage of cell proliferation at the different cell ratios in representative samples from a healthy control subject and an RA patient. **B–D,** Left, Cumulative data show the percentage of proliferating cells **(B)** or levels of cytokines **(C** and **D)** in the absence or presence of Treg cells from healthy controls (n = 8) and patients with RA (n = 8–9). Right, The percentage suppression of Teff cell proliferation is shown for each Teff cell:Treg cell ratio. Bars show the mean ± SEM. The broken horizontal line in **C** and **D** indicates the lower limit of detection. Statistical analysis was performed using Kruskal‐Wallis test with Dunn's multiple comparison test. **∗** = *P* < 0.05; **∗∗** = *P* < 0.01; **∗∗∗** = *P* < 0.001 versus absence of Treg cells. NS = not significant (see Figure 1 for other definitions).

Interestingly, when Treg cells were analyzed in paired PB and SF samples from patients with RA, we observed an increase in the frequency of CD45RO+ memory Treg cells in the SF compared to the PB, and this finding showed a positive correlation with the DAS28 (Spearman's ρ = 0.7988, *P* = 0.0072; n = 10) (Figure 1C). In contrast, the frequency of CD45RO+ Treg cells in the PB did not correlate with the DAS28 (Spearman's ρ = 0.02, *P* = 0.9; n = 41) (results not shown).

Taken together, these data indicate that both the frequency and phenotype of naive and memory Treg cells in the PB were similar between RA patients and healthy controls. Memory Treg cells were, however, increased at the site of inflammation, i.e., in the SF relative to the PB, in patients with RA, and the frequency of synovial Treg cells correlated positively with the level of disease activity.

### Cytokine profiles of PB Treg cells from RA patients and healthy controls

We investigated whether PB‐derived Treg cells in RA patients showed a more proinflammatory cytokine profile, by determining the percentages of IL‐17– and TNF‐expressing Treg cells ex vivo. We focused on memory Treg cells, since this population is more plastic compared to the naive Treg cell population 39. No significant differences in the percentages of IL‐17+ or TNF+ cells within the CD3+CD4+CD45RO+FoxP3+ T cell population were found between PB samples from RA patients and those from healthy controls (Figure 1D).

To investigate whether CD45RO+ Treg cells from RA patients would show increased cytokine expression following in vitro culture with CD14+ monocytes, CD45RO+ Treg cells were sorted from the PB of RA patients (n = 13) and healthy controls (n = 10) and cultured in vitro with autologous CD14+ monocytes (at a 1:1 ratio) in the presence of anti‐CD3 mAb. No significant differences in the percentages of cytokine‐expressing Treg cells (expressing IL‐17, IFNγ, TNF, or IL‐10) were observed between RA patients and healthy controls (see Supplementary Figure 3A, available on the *Arthritis & Rheumatology* web site at http://onlinelibrary.wiley.com/doi/10.1002/art.39408/abstract).

We recently showed that LPS stimulation of CD14+ monocytes results in increased secretion of IL‐1β, IL‐6, and TNF by the monocytes, which thereby drives the expression of IL‐17 by Treg cells 8. However, the addition of LPS to the above cultures did not reveal an increased propensity of Treg cells from RA patients to express the proinflammatory cytokines IL‐17, IFNγ, or TNF as compared to Treg cells from healthy controls (see Supplementary Figure 3B, available on the *Arthritis & Rheumatology* web site at http://onlinelibrary.wiley.com/doi/10.1002/art.39408/abstract). Taken together, these data indicate that CD45RO+ memory Treg cells in the PB of patients with RA do not show an enhanced potential to produce proinflammatory cytokines.

### Treg cell suppression of T cell proliferation, as well as IFNγ and TNF secretion, in autologous Teff cell–monocyte cocultures from patients with RA

We next investigated whether CD45RO+ Treg cells from RA patients are capable of suppressing the proliferation of Teff cells. CD45RA−CD45RO+ Treg cells were sorted from the PB of RA patients (n = 9) and healthy controls (n = 8). Autologous FACS‐sorted CD4+CD45RA−CD45RO+CD25−CD127+ cells (Teff cells) were labeled with CFSE and cocultured with autologous monocytes (at a 1:1 ratio) in the presence of anti‐CD3 mAb. CD45RO+ Treg cells were added to the cultures at 3 different cell ratios (as indicated in Figures 2A–D), and Teff cell proliferation was assessed on day 3 by gating on CFSE+ Teff cells. The presence of CD45RO+ Treg cells, both from RA patients and from healthy controls, led to a cell ratio–dependent reduction in the proliferation of autologous Teff cells (Figures 2A and B). Although in cocultures at the 1:0.5 and 1:1 cell ratios, the mean percentage of Treg cell–mediated suppression was slightly lower in RA PB, this was not significantly different from that in healthy control PB (Figure 2B). The addition of LPS during coculture, to mimic a proinflammatory environment, did not abrogate Treg cell–mediated suppression of Teff cell proliferation in either RA patients or healthy controls (results not shown).

CD45RO+ Treg cells from RA patients were also capable of suppressing the secretion of IFNγ and TNF in these cocultures (Figures 2C and D). Treg cells from some patients appeared to be less efficient in suppressing cytokine secretion, especially at the lower cell ratio (1:0.5); however, the level of suppression was not significantly different from that mediated by Treg cells from healthy controls. IL‐17 secretion was not detectable in any of the conditions.

Interestingly, patients whose PB samples showed less efficient Treg cell–mediated suppression of Teff cell proliferation were not necessarily those whose PB samples showed less efficient suppression of cytokine production (e.g., RA patient 5 in Supplementary Table 1, available on the *Arthritis & Rheumatology* web site at http://onlinelibrary.wiley.com/doi/10.1002/art.39408/abstract). Furthermore, less efficient Treg cell–mediated suppression of Teff cell proliferation could also be observed in the PB of certain healthy control subjects (e.g., healthy controls 5 and 6 in Supplementary Table 1). We did not find an association between clinical parameters (DAS28, CRP level, ESR, disease duration) and the ability of Treg cells from RA patients to suppress proliferation. Notably, Treg cells from the patient with the highest DAS28 were among the better suppressors (see RA patient 9 in Supplementary Table 1).

It was shown that in patients with multiple sclerosis, a functional defect resides within naive Treg cells 40, 41. We found that CD45RA+ Treg cells from both RA patients and healthy controls suppressed less efficiently than did their CD45RO+ Treg cell counterparts. However, the overall ability of CD45RA+ Treg cells to suppress CD45RO+ Teff cell proliferation (at a 1:1 ratio of Teff cells to Treg cells) was not significantly different between RA patients (mean ± SD 21 ± 8% suppression; n = 7) and healthy controls (33 ± 8% suppression; n = 6) (*P* = 0.37) (see Supplementary Table 1).

**Figure 3:**
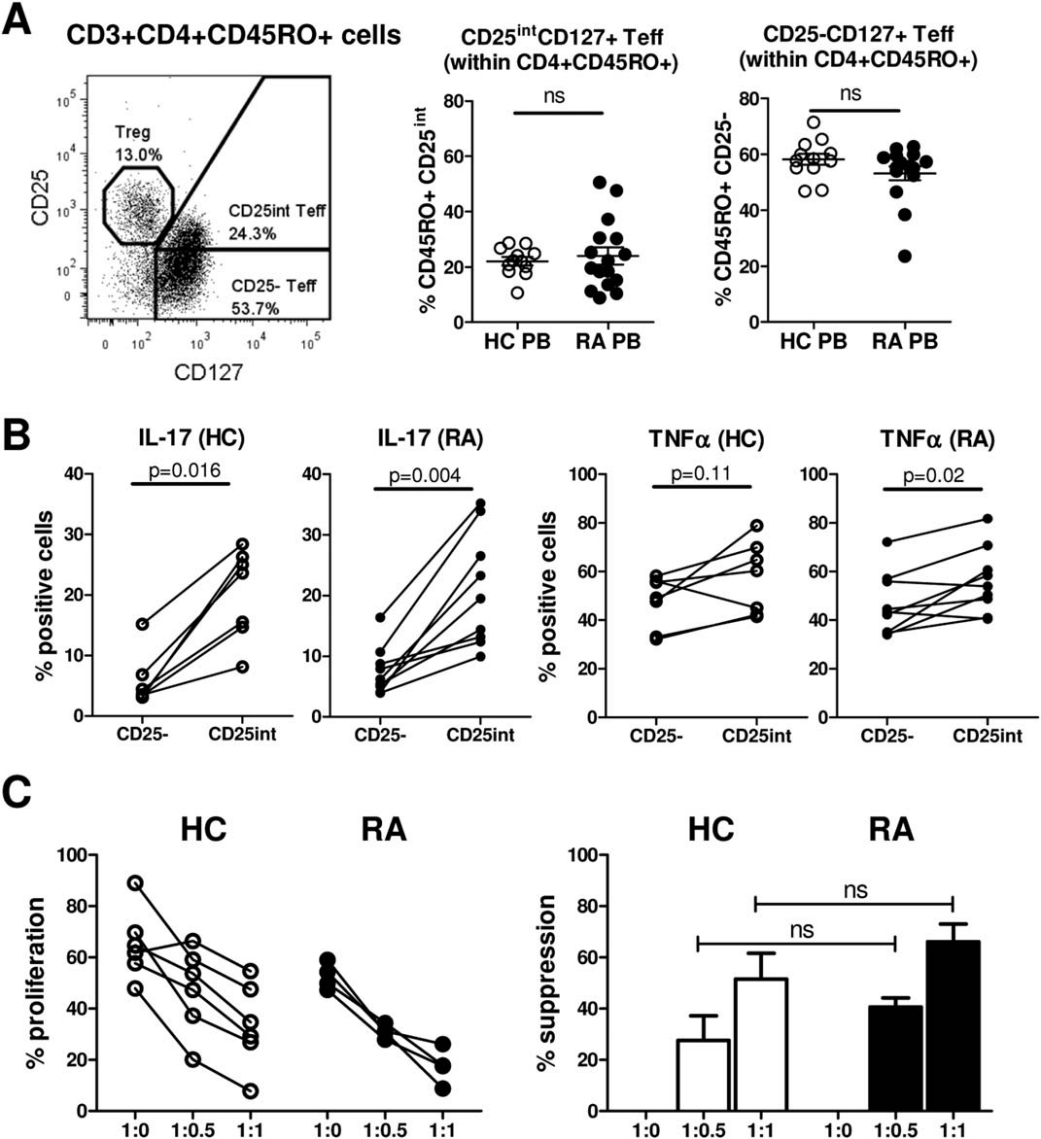
Ability of CD45RO+ Treg cells from patients with RA to suppress CD25^intermediate^ (CD25^int^) Teff cell proliferation and cytokine production. **A,** The percentage of CD25^int^CD127+ and CD25−CD127+ Teff cells within CD4+CD45RO+ T cells was determined in PB samples from healthy controls (n = 12) and RA patients (n = 16). Symbols represent individual donors; horizontal bars show the mean. Groups were compared by unpaired *t*‐test. **B,** CD25−CD127+ and CD25^int^CD127+ Teff cells were sorted from CD4+CD45RO+ T cells in the PB of healthy controls (n = 7) and RA patients (n = 9) and cocultured with autologous monocytes in the presence of anti‐CD3. The percentages of Teff cells producing IL‐17 or TNF were determined following restimulation of the cells with phorbol myristate acetate and ionomycin on day 3. Data were analyzed by Wilcoxon's matched pairs signed rank test. **C,** CD4+CD45RA−CD45RO+CD25^int^CD127+ Teff cells from healthy controls and RA patients were labeled with 5,6‐carboxyfluorescein succinimidyl ester and cocultured with CD14+ monocytes at a 1:1 cell ratio in the presence of anti‐CD3 monoclonal antibody (100 ng/ml), in the absence or presence of autologous sorted CD45RO+CD25+CD127^low^ Treg cells (added at the indicated Teff cell:Treg cell ratios). On day 3, Teff cell proliferation was assessed by flow cytometry. Left, Cumulative data show the percentage of proliferating cells in the absence or presence of Treg cells from healthy controls (n = 6) and RA patients (n = 4). Right, The percentage suppression of Teff cell proliferation is shown for each Teff cell:Treg cell ratio. Bars show the mean ± SEM. Data were analyzed by Mann‐Whitney test. NS = not significant (see Figure 1 for other definitions).

Recently, it was shown that the ability of Treg cells to suppress proliferation of Teff cells with intermediate CD25 expression (CD25^int^ Teff cells) was reduced compared to their ability to suppress proliferation of CD25− Teff cells. This could be attributed to a more activated phenotype and higher proliferation rate of CD25^int^ Teff cells 42. We found that the frequencies of CD25^int^ Teff cells were similar between the PB of RA patients and the PB of healthy controls (Figure 3A). In both RA patients and healthy controls, CD25^int^ Teff cells, compared to CD25− Teff cells, contained higher percentages of IL‐17–expressing and TNF‐expressing cells following stimulation (Figure 3B), indicating that the CD25^int^ Teff cell population may contain more activated cells. Treg cells from RA patients were, however, able to effectively suppress the proliferation of autologous CD25^int^ Teff cells, similar to the findings in healthy controls (Figure 3C). Taken together, these data show there is no significant difference in the ability of Treg cells to suppress autologous Teff cell proliferation or the secretion of IFNγ or TNF in monocyte–T cell cocultures from the PB of RA patients compared to that from healthy controls.

### Suppression of monocyte‐derived cytokines and chemokines by Treg cells from RA patients

**Figure 4:**
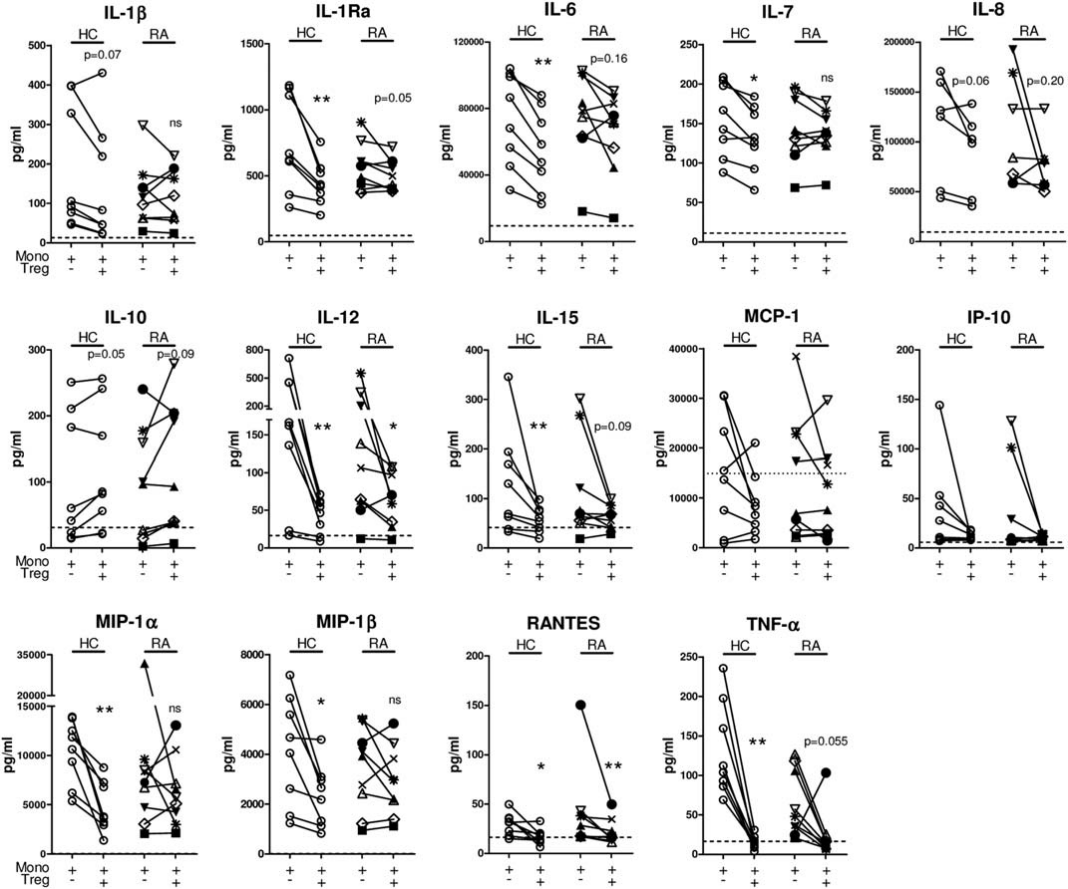
Ability of CD45RO+ Treg cells from patients with RA to suppress lipopolysaccharide (LPS)–induced monocyte (Mono)–derived cytokine/chemokine production. CD14+ monocytes from healthy controls (n = 6–8) and RA patients (n = 7–9) were cultured in the presence of 100 ng/ml LPS without or with sorted CD45RO+ Treg cells at a 1:1 ratio. After 3 days, the supernatants were collected and analyzed using a human 25‐plex cytokine array or by enzyme‐linked immunosorbent assay (for IL‐6 and IL‐8). Each line joined by symbols represents an individual donor. The broken horizontal line indicates the lower limit of detection. For monocyte chemotactic protein 1 (MCP‐1), the dotted horizontal line indicates the upper limit of detection. Data were analyzed by Wilcoxon's matched pairs signed rank test. **∗** = *P* < 0.05; **∗∗** = *P* < 0.01 versus absence of Treg cells. IL‐1Ra = interleukin‐1 receptor antagonist; IP‐10 = interferon‐γ–inducible protein 10; MIP‐1α = macrophage inflammatory protein 1α; NS = not significant (see Figure 1 for other definitions).

We recently demonstrated that CD14+ PB‐derived monocytes from RA patients show a more activated phenotype compared to PB monocytes from healthy controls 8. We therefore investigated the ability of Treg cells from RA patients to suppress LPS‐induced monocyte‐derived cytokine and chemokine production. PB monocytes from RA patients (n = 9) and healthy controls (n = 8) were cultured with LPS in the absence or presence of autologous CD45RO+ Treg cells for 3 days. LPS‐activated PB monocytes from RA patients and those from healthy controls produced similar levels of cytokines/chemokines in the absence of Treg cells (Figure 4). In the presence of Treg cells, although considerable interdonor differences were observed, similar overall levels of suppression were found in the majority of RA patients and healthy controls with regard to suppression of IL‐6, IL‐8/CXCL8, IL‐12, IL‐15, IFNγ‐inducible protein 10/CXCL10, RANTES/CCL5, and TNF. Although a lack of suppression of individual cytokines/chemokines was observed in some patients (e.g., IL‐8 in a patient with RA [open downward triangles in Figure 4]), Treg cells from these same patients efficiently suppressed the other cytokines/chemokines.

More notable differences were observed with regard to Treg cell suppression of IL‐1β, IL‐1 receptor antagonist (IL‐1Ra), IL‐7, macrophage inflammatory protein 1α (MIP‐1α)/CCL3, and MIP‐1β/CCL4, which were suppressed by CD45RO+ Treg cells from healthy controls, whereas this was not consistently observed in cultures with Treg cells from RA patients (Figure 4 and Supplementary Table 2, available on the *Arthritis & Rheumatology* web site at http://onlinelibrary.wiley.com/doi/10.1002/art.39408/abstract). However, again this was not a global defect, as PB samples from ∼44% of the RA patients did show an intact ability to suppress these chemokines and cytokines. Monocyte chemotactic protein 1 was not significantly suppressed by Treg cells from either RA patients or healthy controls, and IL‐10 was mostly increased in the presence of Treg cells both from RA patients and from healthy controls, as has been previously shown in healthy individuals 43. Taken together, these data indicate that some differences can be observed in the capacity of Treg cells from RA patients to suppress secretion of certain cytokines/chemokines by LPS‐activated monocytes. However, this impairment was not detected for all cytokines/chemokines and, furthermore, was not observed in all patients.

### Gene expression profiles of PB CD45RA+ and CD45RO+ Treg cells and Teff cells from RA patients and healthy controls

**Figure 5:**
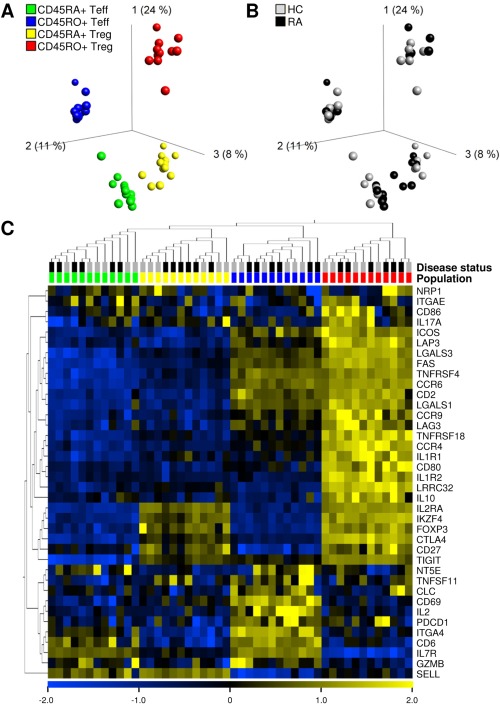
Gene expression profiling of CD45RA+ and CD45RO+ Treg and Teff CD4+ T cell subsets from the peripheral blood (PB) of healthy controls (HC) and patients with rheumatoid arthritis (RA). CD25+CD127^low^ Treg cells and CD25−CD127+ Teff cells were sorted from the CD45RA+CD45RO− (naive) or CD45RA−CD45RO+ (memory) CD4+ T cell compartments from the PB of age‐ and sex‐matched healthy controls (mean age 54 years [range 34–69]) and RA patients (mean age 57 years [range 37–75]) (each n = 6 female donors). Five of the RA patients were receiving disease‐modifying antirheumatic drug treatment (n = 3 methotrexate [MTX], n = 1 hydroxychloroquine [HCQ], n = 1 MTX, HCQ, and sulfasalazine); 1 patient had received no medication. All patients had moderate‐to‐active disease, with a mean ± SEM Disease Activity Score in 28 joints of 4.8 ± 0.3. **A,** Principal components analysis (PCA) based on global gene expression levels in the PB shows distinct clusters of the CD45RA+ Teff, CD45RA+ Treg, CD45RO+ Teff, and CD45RO+ Treg cell populations. **B,** PCA shows that the 4 T cell populations do not cluster differently between healthy controls and RA patients. **C,** The heatmap, representing results from hierarchical cluster analysis, shows the relative expression levels of previously described Treg signature–associated genes in the different T cell subsets from the PB of healthy controls and RA patients.

Finally, we assessed whether Treg cells from RA patients are different at a molecular level, using genome‐wide gene expression profiling of FACS‐sorted PB‐derived Treg cells from the CD45RA+CD45RO− (naive) and CD45RO+CD45RA− (memory) T cell compartments from the PB of RA patients and healthy controls. CD45RA+ and CD45RO+ Teff cells were isolated in parallel. As shown by principal components analysis, each of the 4 CD4+ T cell subsets was found to be grouped as a distinct cluster based on global messenger RNA expression levels (Figure 5A). However, cells from RA patients did not cluster separately from cells derived from healthy controls, within either the Treg cell or Teff cell populations (Figure 5B). In fact, no significant differentially expressed genes were found when comparing RA cells and healthy control cells in any of the 4 populations (false discovery rate 5%, fold change in gene expression 1.5).

To verify that the sorted Treg cells were truly of a regulatory cell type, we investigated the relative expression levels of genes previously associated with a Treg cell phenotype 44. As visualized in the heatmap shown in Figure 5C, CD45RO+ Treg cells as well as CD45RA+ Treg cells could be clearly distinguished from their Teff cell counterparts based on the expression of well‐described Treg signature genes, including expression of *FOXP3*, *CTLA4*, *CD25*, *CD27*, and *CD127* (Figure 5C). In summary, the ex vivo gene expression profiles of Treg cells from the PB of RA patients and healthy controls were highly similar, supporting the other observations made herein showing that Treg cells in the PB of RA patients are not intrinsically different from those in the PB of healthy individuals.

## DISCUSSION

In this report we have presented a comprehensive analysis of the frequency, phenotype, cytokine profile, suppressive potential, and gene expression profile of CD4+CD25+CD127^low^ Treg cells from both the CD45RO+ and CD45RA+ T cell compartments of the PB from patients with established RA compared to age‐ and sex‐matched healthy individuals. Collectively, our findings indicate that CD4+CD25+CD127^low^ Treg cells in the blood of patients with chronic RA are not intrinsically or globally defective.

We show that PB Treg cell frequencies are similar in RA patients and healthy controls in both the CD45RO+ and CD45RO− T cell compartments, thus extending the findings from previous studies 1, 3, 4, 17. Consistent with the results reported in earlier studies 1, 2, 3, 4, 5, 6, 7, 8, 25, we found that the percentages of CD45RO+ Treg cells were significantly increased in the RA SF compared to paired RA PB samples, and notably, this positively correlated with the DAS28. Phenotypically, PB Treg cells showed similar expression levels of CD4, CD25, CD127, CD39, and CD161 in RA patients and healthy controls, with a slight, but significantly, increased expression of FoxP3 in RA patients, both in CD45RO+ Treg cells and in CD45RO− Treg cells.

Recent data indicate that Treg cells, particularly those with a memory phenotype, can convert into IL‐17+ Treg cells under proinflammatory conditions (for review, see ref. 45). However, our data showed that CD45RO+ Treg cells from RA patients did not contain significantly enhanced percentages of IL‐17+, IFNγ+, or TNF+ cells, when analyzed ex vivo or after in vitro culture. Although some groups have reported that Treg cells from the blood of RA patients are unable to suppress Teff cell proliferation 14, 15, 17, the majority of studies reporting on the suppressive function of PB‐derived Treg cells in RA have demonstrated intact Treg cell function when the suppression of Teff cell proliferation was assessed 2, 5, 7, 13, 23, 24. Consistent with these observations, our findings also indicated that in RA patients, PB‐derived Treg cells from either the CD45RO+ or the CD45RA+ T cell compartment were functionally intact with regard to suppression of Teff cell proliferation. In addition, we demonstrated that Treg cells from RA patients can suppress proliferation of both CD25− and CD25^int^ Teff cells to a similar extent as healthy control Treg cells, even though CD25^int^ Teff cells contained increased percentages of IL‐17+ and TNF+ cells, suggesting a potentially higher activation status of these cells.

Several groups have reported that in RA, PB‐derived Treg cells are defective in their ability to suppress IFNγ and/or TNF production 13, 14, 15, 16, 17, whereas others have reported that Treg cells have intact suppressive function 2, 3, 7. Our current data indicate that the ability of Treg cells from the blood of RA patients to suppress these cytokines is not significantly impaired. It has been suggested that Treg cells from RA patients are impaired in their ability to suppress IL‐17 18, 46. In our assays, IL‐17 levels were below the limit of detection. Analysis of the ability of Treg cells to suppress monocyte‐derived cytokines/chemokines revealed no consistent differences between RA patients and healthy controls with regard to the suppression of TNF, IL‐6, IL‐8, IL‐12, IL‐15, and CCL5, although suppression seemed reduced in some patients. We did find impaired suppression of IL‐1β, IL‐1Ra, IL‐7, CCL3, and CCL4 in RA PB relative to healthy control PB; however, this was not consistently observed in all patients. Importantly, none of the patients displayed impairment in all tested aspects of Treg cell–mediated suppression. Additionally, in healthy controls, some degree of variation in Treg cell suppressive function was observed.

Findings in a recent study suggested that Treg cell suppressive function (defined by suppression of T cell proliferation and IFNγ production) was defective in patients with early, active RA who had not yet received therapy (treatment‐naive), whereas Treg cell suppressive function was not defective in patients with chronic RA who had been exposed to methotrexate (MTX), although the group numbers were small (n = 4 per study group) 47. Another recent study in patients with RA suggested that Treg cell function was reduced in MTX nonresponders, and that this defect was related to low CD39 expression 48. The identification of intact peripheral Treg cell function in patients with chronic RA who had received therapy with disease‐modifying antirheumatic drugs (DMARDs)/biologic drugs, as described herein and in previous studies 2, 7, 23, 24, could thus be a consequence of effective DMARD therapy. However, other studies have shown no defects in the suppressive capacity of CD4+CD25^high^ Treg cells from patients with early, active RA who were being treated with steroids and were DMARD‐naive, as compared to patients who were being treated with DMARDs and had well‐controlled disease or healthy controls 5, 7. In our study, none of the patients had early RA or were treatment‐naive, and therefore further studies are required before conclusive statements can be made regarding Treg cell function in treatment‐naive patients with early RA. Nonetheless, the notion that DMARD or biologic therapy may restore defective Treg cell function is important to consider.

It is also possible that disease activity influences Treg cell function. However, when we compared the results with regard to suppression of IFNγ and TNF production and T cell proliferation between those patients with highly active disease (DAS28 >5.1; n = 4) and those with moderate disease activity (DAS28 3.2–5.1; n = 4) (see Supplementary Table 1), we observed neither considerable differences nor consistent differences between these 2 groups with respect to Treg cell function. This finding clearly indicates that even in patients with active disease, Treg cells are still functional. Taken together with our finding that Treg cells are increased in the inflamed joint and this is correlated with the DAS28, it is reasonable to suggest that Treg cell activity is preserved, and may even be enhanced 8, during inflammation in chronic RA, as a means to dampen collateral damage. This is supported by experimental models showing that Treg cells are increased in number and function during inflammation 49 and that TNF can boost Treg cell function 50.

The notion that PB‐derived Treg cells are not defective in patients with chronic RA is strengthened by our findings that neither CD45RA+ nor CD45RO+ Treg cells displayed significantly differentially expressed genes relative to their counterparts from the PB of sex‐ and age‐matched healthy controls. One limitation of in vitro suppression assays is that cells are taken out of their inflammatory environment when tested for suppressive activity. Our genome‐wide expression arrays were performed on cells that were isolated from patients directly ex vivo, thus closely resembling the in vivo situation. An interesting side observation from our work was that a large proportion of Treg signature genes are predominantly expressed in CD45RO+, but not CD45RA+, Treg cells, suggesting that current investigations of Treg cell–specific signature genes may be biased toward features of the memory Treg cell population.

Importantly, our findings do not exclude the probability that cues from the local inflammatory environment can impede Treg cell–mediated suppression. Indeed, inflammatory cytokines like IL‐6 and TNF can negatively modulate FoxP3 activity 14, 51. A recent study showed that PB‐derived Treg cells from RA patients were unable to suppress Teff cell proliferation or TNF or IFNγ secretion by autologous PB Teff cells, and this was attributed to the increased resistance of Teff cells to Treg cell–mediated suppression, rather than to defective Treg cell function 31. Increased resistance of Teff cells to regulation was also reported in a spontaneous experimental arthritis model 49. Furthermore, using crossover experiments, it was shown that activated T cells from the inflamed joints of patients with RA were more resistant to Treg cell–mediated suppression than their PB counterparts 2, 26, 28, 29, 30.

Collectively, our data indicate that Treg cells from the PB of patients with chronic RA are not intrinsically different from those in healthy individuals. Combined with previous studies showing intact suppressive function of Treg cells derived from the inflamed joint 1, 2, 3, 4, 5, 6, 7, 25, 26, 27, these findings imply that persistent disease activity in chronic RA cannot simply be attributed to defective CD4+CD25+CD127^low^ Treg cell function in the circulation.

## AUTHOR CONTRIBUTIONS

All authors were involved in drafting the article or revising it critically for important intellectual content, and all authors approved the final version to be published. Dr. Taams had full access to all of the data in the study and takes responsibility for the integrity of the data and the accuracy of the data analysis.

### Study conception and design

Walter, Frederiksen, Evans, Taams.

### Acquisition of data

Walter, Frederiksen, Rajasekhar, Menon, Evans.

### Analysis and interpretation of data

Walter, Fleskens, Frederiksen, Rajasekhar, Menon, Gerwien, Evans, Taams.

## ADDITIONAL DISCLOSURES

Authors Frederiksen and Gerwien are employees of Novo Nordisk.

## Supporting information

Supporting Information Figure 1

Supporting Information Figure 2

Supporting Information Figure 3

Supporting Information Table 1

Supporting Information Table 2
