# Supplementary figures and images for: Phenotypic, Functional, and Gene Expression Profiling of Peripheral CD45RA+ and CD45RO+ CD4+CD25+CD127low Treg Cells in Patients With Chronic Rheumatoid Arthritis
Source: Arthritis Rheumatol. 2015 Dec 23;68(1):103–16. doi: 10.1002/art.39408 (PMC4832388; doi:10.1002/art.39408)

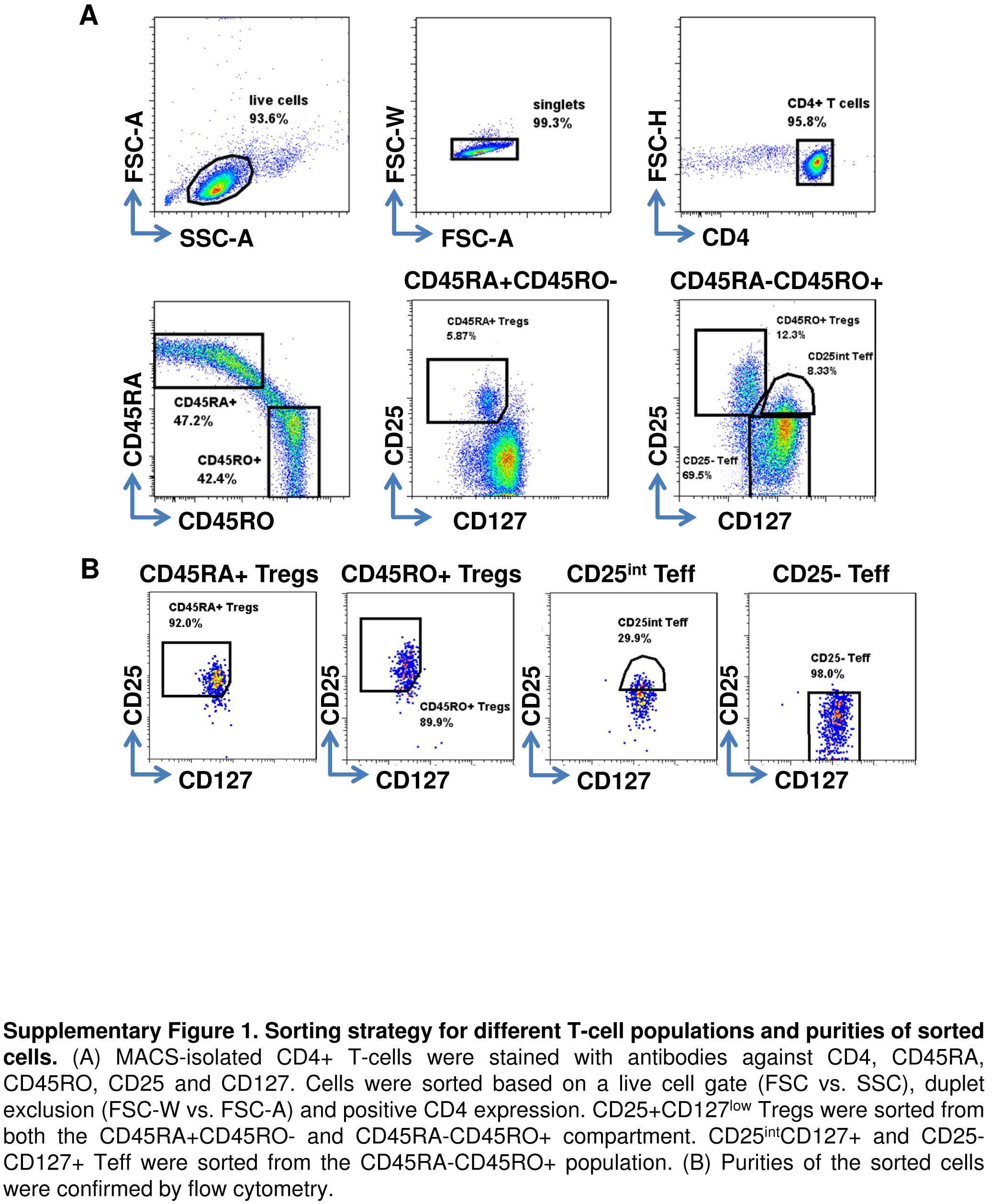

Supplement: Supplementary file 1 — Supporting Information Figure 1 [file ART-68-103-s001.tif]

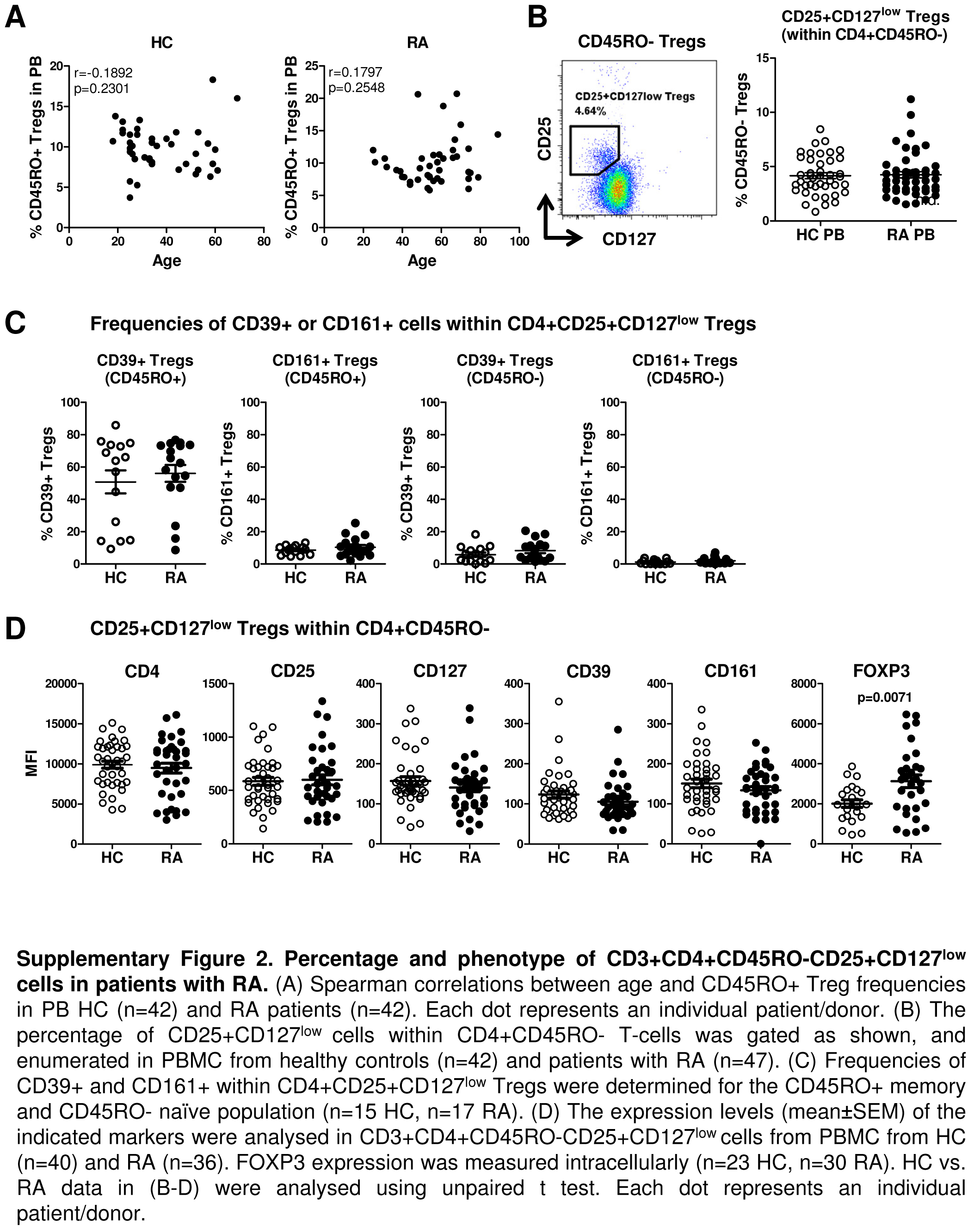

Supplement: Supplementary file 2 — Supporting Information Figure 2 [file ART-68-103-s002.tif]

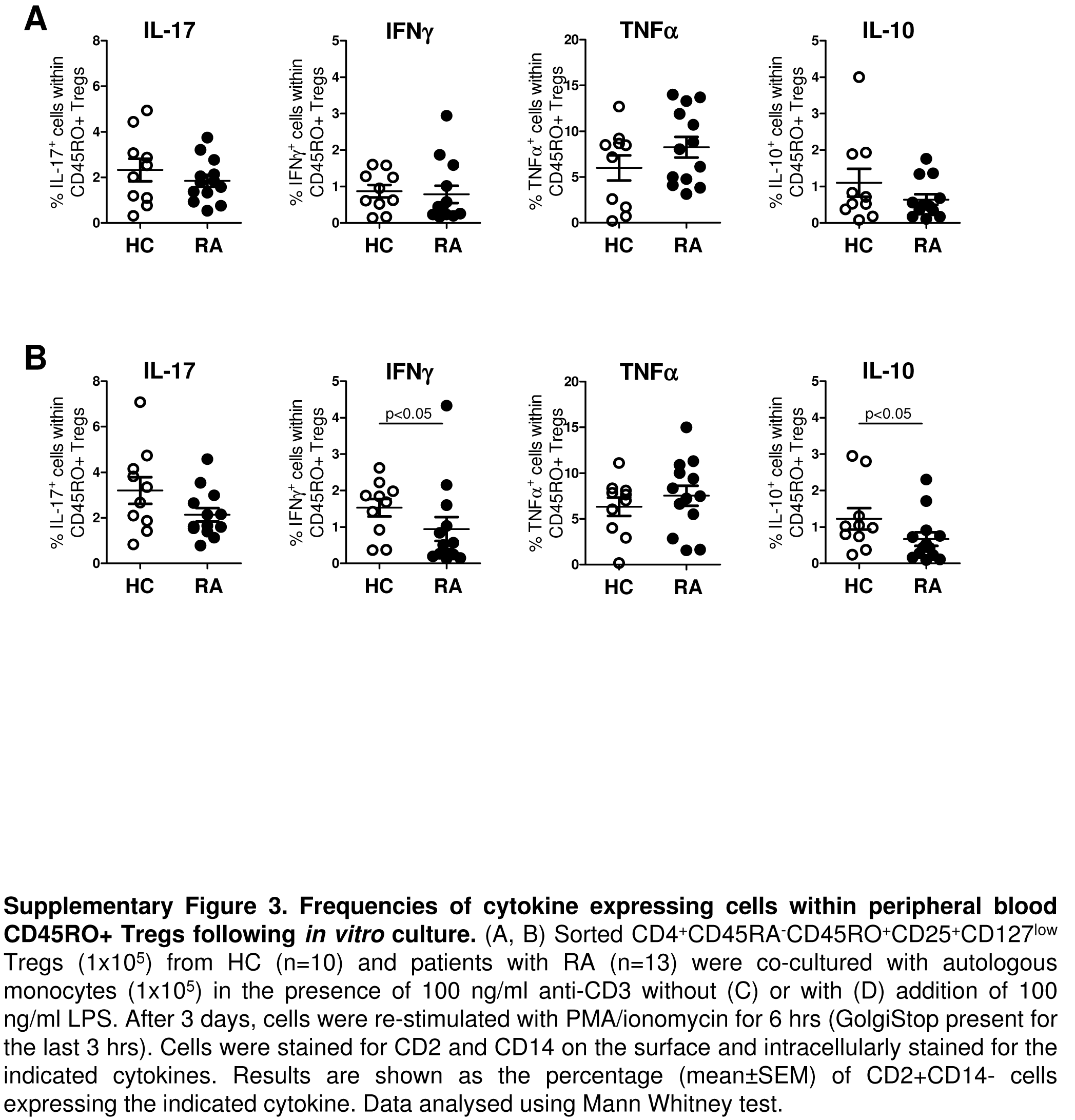

Supplement: Supplementary file 3 — Supporting Information Figure 3 [file ART-68-103-s003.tif]

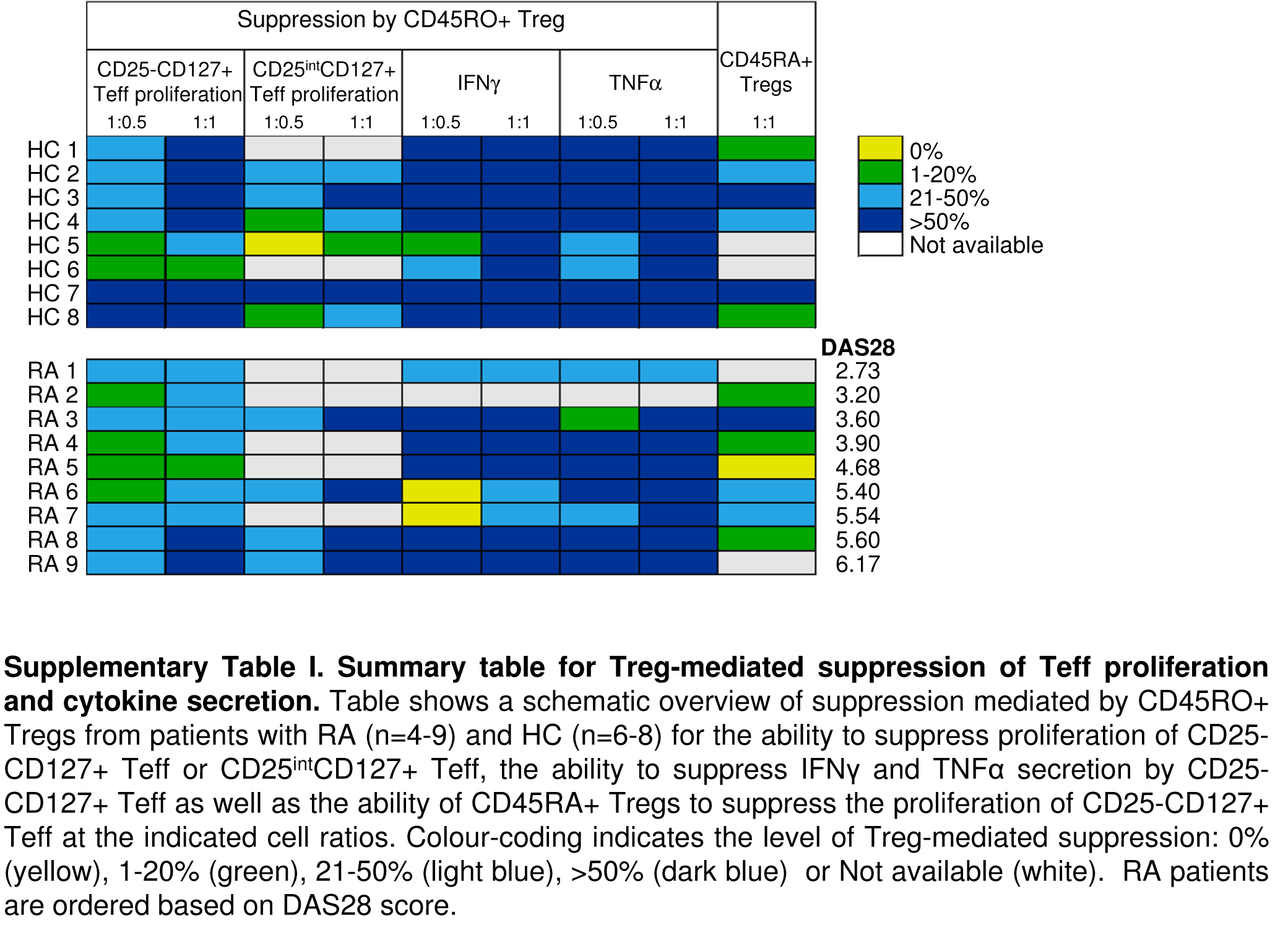

Supplement: Supplementary file 4 — Supporting Information Table 1 [file ART-68-103-s004.tif]

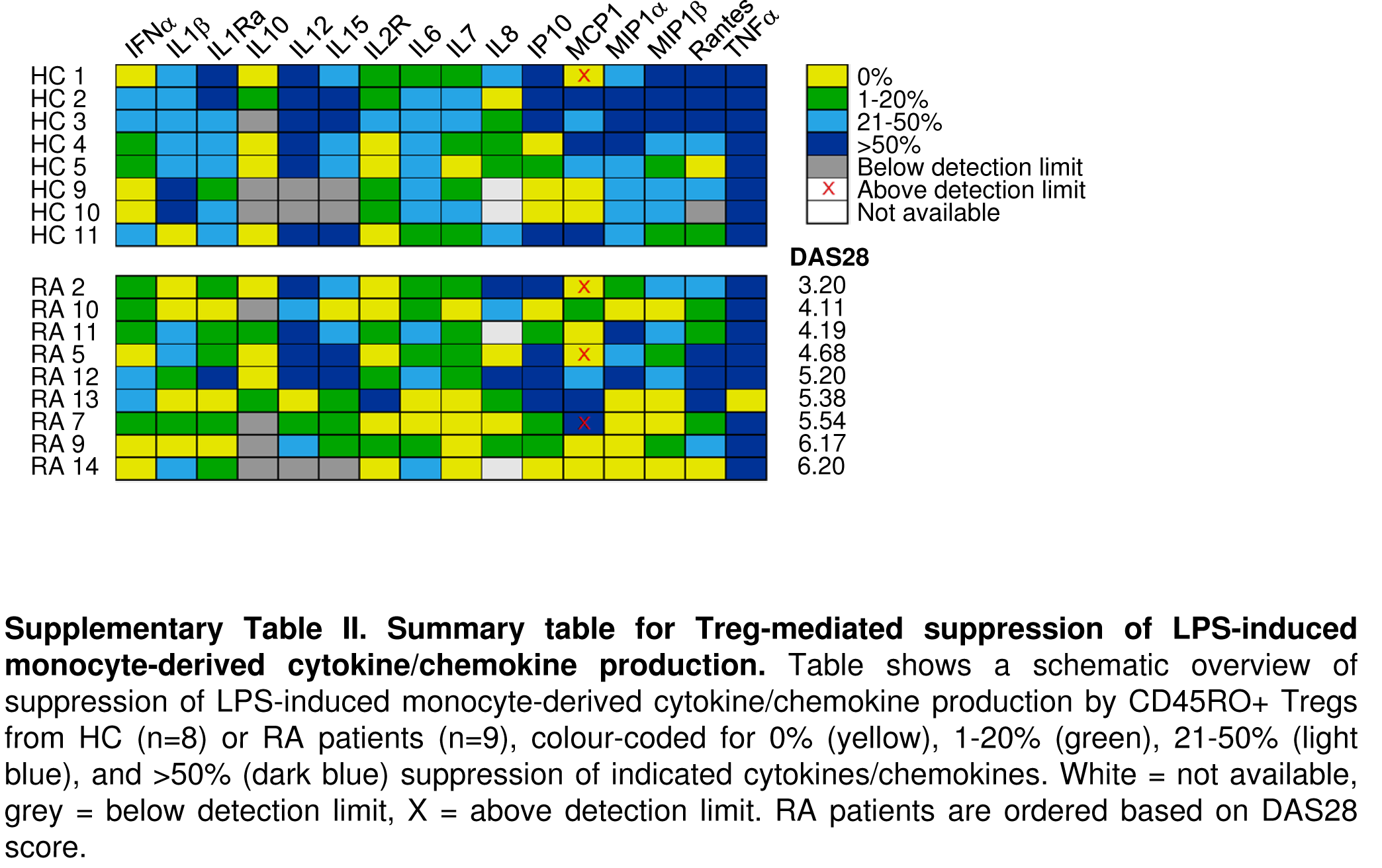

Supplement: Supplementary file 5 — Supporting Information Table 2 [file ART-68-103-s005.tif]
